# Physicians' views on resource availability and equity in four European health care systems

**DOI:** 10.1186/1472-6963-7-137

**Published:** 2007-08-31

**Authors:** Samia A Hurst, Reidun Forde, Stella Reiter-Theil, Anne-Marie Slowther, Arnaud Perrier, Renzo Pegoraro, Marion Danis

**Affiliations:** 1 Institute for Biomedical Ethics, Geneva University Medical School, Switzerland; 2 The Research Institute, Norwegian Medical Association and University of Oslo, Norway; 3 Institute for Applied Ethics and Medical Ethics, University of Basel, Switzerland; 4 The Ethox Centre, Oxford University, Headington, UK; 5 General Internal Medicine Service, Geneva University Hospital, Geneva, Switzerland; 6 Fondazione Lanza, Padova, Italy; 7 National Institutes of Health, Bethesda, MD, USA

## Abstract

**Background:**

In response to limited resources, health care systems have adopted diverse cost-containment strategies and give priority to differing types of interventions. The perception of physicians, who witness the effects of these strategies, may provide useful insights regarding the impact of system-wide priority setting on access to care.

**Methods:**

We conducted a cross-sectional survey to ascertain generalist physicians' perspectives on resources allocation and its consequences in Norway, Switzerland, Italy and the UK.

**Results:**

Survey respondents (N = 656, response rate 43%) ranged in age from 28–82, and averaged 25 years in practice. Most respondents (87.7%) perceived some resources as scarce, with the most restrictive being: access to nursing home, mental health services, referral to a specialist, and rehabilitation for stroke. Respondents attributed adverse outcomes to scarcity, and some respondents had encountered severe adverse events such as death or permanent disability. Despite universal coverage, 45.6% of respondents reported instances of underinsurance. Most respondents (78.7%) also reported some patient groups as more likely than others to be denied beneficial care on the basis of cost. Almost all respondents (97.3%) found at least one cost-containment policy acceptable. The types of policies preferred suggest that respondents are willing to participate in cost-containment, and do not want to be guided by administrative rules (11.2%) or restrictions on hospital beds (10.7%).

**Conclusion:**

Physician reports can provide an indication of how organizational factors may affect availability and equity of health care services. Physicians are willing to participate in cost-containment decisions, rather than be guided by administrative rules. Tools should be developed to enable physicians, who are in a unique position to observe unequal access or discrimination in their health care environment, to address these issues in a more targeted way.

## Background

Limited resources are a reality to which health care systems respond in very different ways. As physicians are confronted with scarcity and with the effects of cost-containment policies on clinical practice, they occupy a unique position from which to observe the impact of priorities set by health care systems.

Contradictory data exist as to whether physicians are aware of facing scarcity. In *The Painful Prescription: Rationing Hospital Care*, Aaron and Schwartz noted that British physicians rationalized, or redefined health care standards to face scarcity more comfortably. [1] Twenty years later, researchers conducting interviews with physicians regarding scarcity reported being struck with the strength with which scarcity was denied. [2] US general internists, intensive care specialists, and oncologists, however, do report difficulties explicitly associated with resource scarcity. [3] Data suggest that physicians accept prioritization decisions, both when faced with hypotheotical scenarios, [4-13] and when reporting on their practice. [3,14-16] Physicians at the point of care are uniquely situated to observe the impact of priority setting decisions on patients in the form of scarcity, or less than equitable care. Their experience may thus yield useful insights and feedback about the impact of priorities on clinical care, which could contribute to evidence-based health policy. [17] Despite this, insufficient attention is paid to their experience.

To examine the perceptions and attitudes of physicians regarding resource allocation in the European context, we conducted a three-part international survey of general physicians in Italy, Norway, Switzerland, and the UK. Results from the two other parts of this survey have been reported elsewhere. [16,18] In this paper, we report physicians' perception regarding lack of resource availability in their health care system and its adverse effects, their views regarding the equity of their health care system, and their attitudes towards various cost-containment policies.

## Methods

### Participants

General physicians were identified through the 2002 official list of the Norwegian Medical Association, the Swiss Medical Association, published listings of UK general practitioners and general physicians, and regional listings of Italian general practitioners and members of the Italian Society of Internal Medicine. A random sample of 400 individuals was drawn in each country in proportions of general practitioners and general internists reflecting that of each national physician population. This sample was chosen to capture similar physician populations, who do the same kind of work in general internal medicine, in both in- and outpatient care. We chose four European countries offering universal access to health care through very different systems, with per capita expenditure on health care ranging from $3,322 in Switzerland to $1,989 in the UK (2002 US $). Despite differences in structure and health care expenditure, the health care systems of all four countries received similar evaluations regarding fairness of financial contribution to the health system and distribution of responsiveness in the WHO world health report of 2000 (Table 1).

**Table 1:** Four Health Care Systems: WHO and OECD data

***Per capita expenditure on health care***^*a*^	**Italy**	**Norway**	**Switzerland**	**UK**
				
Total (2002 US $)	2,166	3,409	3,446	2,160
Public (2002 US $)	1,639	2,845	1,995	1,801
Out of pocket (2002 US$)	440	546	1,085	200
				
***Proportion of expenditure on health care***^*a*^				
Social security	0.1%	0%	40%	0%
Other public	75.5%	83.5%	17.9%	83.4%
Pre-paid plan	1%	0%	9.6%	3%
Out of pocket	20.3%	16%	31.4%	9.2%
Other private	3%	0.5%	1.4%	4.3%
				
***Beds, physicians, nurses***^*b*^				
Acute care beds/1000 p.	3.7	3.1	3.9	3.7
Nursing home beds/1000 p.	2.7	9.1	11.6	3.1
Nurses/1000 pop.	5.4	10.4	10.7	9.7
Physicians/1000 pop.	4.1	3.4	3.7	2.2
				
***Elements of health policy***				
Universal coverage	Yes	Yes	Yes	Yes
Freedom to choose general physician	Yes	Yes	Yes	No
Gatekeeping for specialist consultation	Yes	Yes	No	Yes
				
***WHO assessment of equity***^*c*^				
Fairness of financial contribution to health system	0.961	0.977	0.964	0.977
Distribution of responsiveness	0.995	0.995	0.995	0.995

a WHO 2002 country information [40]

b OECD 2003 country information [33]

c WHO 2000 World health report [41]

### Survey methods

We developed a survey instrument to explore general physicians' perception of scarcity and rationing both at the system-wide level, through resource unavailability, and in clinical practice, through bedside rationing. Whenever possible, we used validated items from other studies published in the literature [14,15,19,20]. This included items relating to agreement with various cost-containment policies [14]. New items were independently rated by two ethicists with relevant expertise. The questionnaire was refined following their comments and piloted on 96 physicians in the US, the UK, and Switzerland. Each scale was tested for internal consistency on the pilot sample, and again on the complete sample. Survey development was further described elsewhere [16].

**Figure 1:**
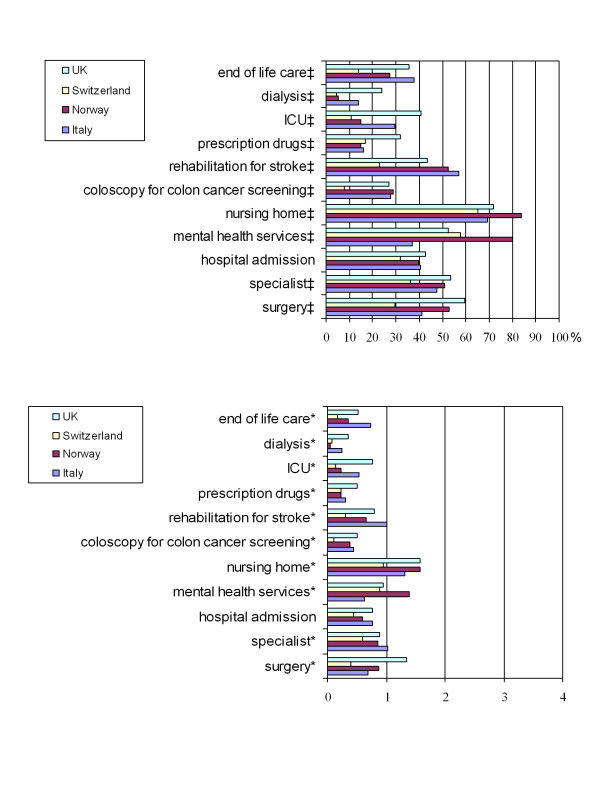
**Limited resources**. During the last six months, how often were you unable to obtain the following services for your patients when you thought they were necessary (this includes unacceptable waiting times)?. **Panel A: Percentage of respondents who reported unavailability of resources**. ‡Chi-square: p < 0.01; null hypothesis is "no difference". **Panel B: mean frequency of reported unavailability of resources**. 0 = "never", 1 = "less than once a month", 2 = "once a month", 3 = "weekly", 4 = "daily". *Kruskall-Wallis: p < 0.01; null hypothesis is "no difference".

A Perceived scarcity scale assessed resource unavailability was worded as follows: "During the last 6 months, how often were you unable to obtain the following services for your patients when you thought they were necessary (this includes unacceptable waiting times)?". It was based on items worded as shown in Figure 1. Response options were: never or not applicable (0), less than once a month (1), monthly (2), weekly (3), and daily (4). Scale range was 0–44. Internal consistency was good with a Crohnbach's alpha of 0.84, range was 0–44 We also asked respondents about pressure to ration and underinsurance using the following items: "In the last six months, how often have you felt under pressure to deny an expensive intervention that you thought was indicated? ", and "In the last six months, how often have you found in your work that patients have problems that cannot be treated because they cannot afford their share of the costs?". These items used the same response options as the perceived scarcity scale.

Physicians' experience regarding adverse effects of scarcity was explored using items worded as follows: "In the last six months, how often have you seen a situation where a patient suffered adverse consequences as a result of limited resources in the health care system?". This item used the same response options as the perceived scarcity scale. A follow-up item asked: "What is the most severe adverse consequence you have seen as a result of limited resources in the health care system?". Response options were: inconvenience, temporary disability, permanent disability, an acute life-threatening event, death, or none.

**Figure 2:**
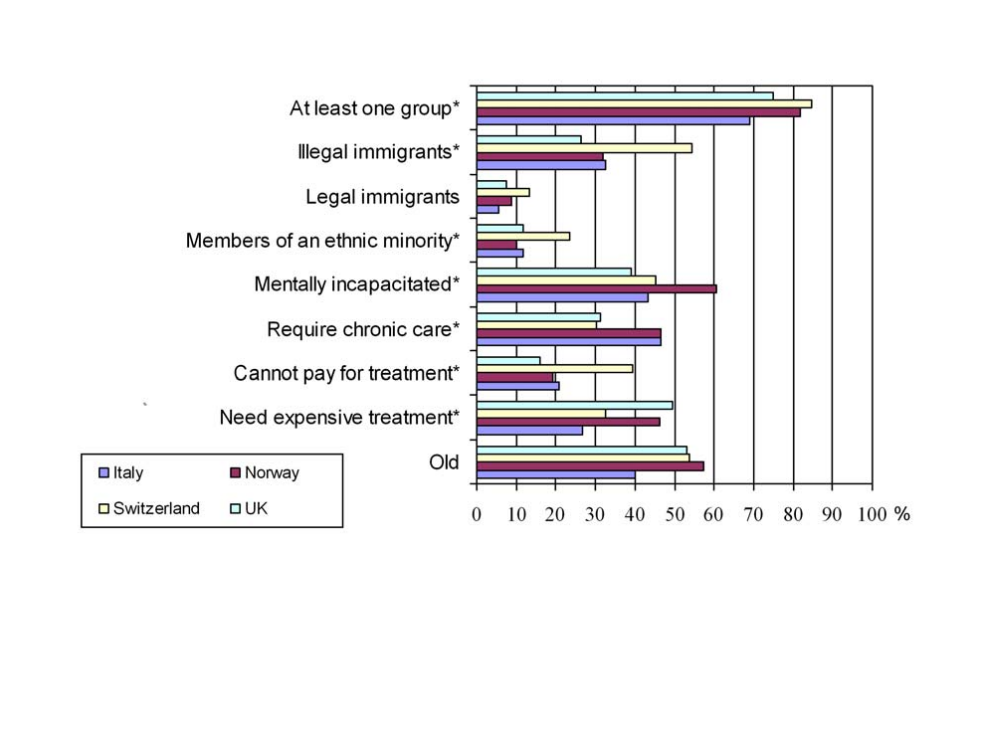
**Reported greater likelihood to be denied treatment based on group identity**. Based on your experience, are patients who belong to any of the following groups more likely than others to be denied beneficial care on the basis of cost in your health care environment?. *Pearson Chi-Square: p < 0.01; null hypothesis is "no difference".

A Perceived equity scale (Cronbach's alpha 0.78, range 3–15) was based on items worded as shown in table 2. Responses were on a 5 point Likert scale ranging from "strongly agree" (5) to "strongly disagree" (1). A Perceived discrimination index was worded as shown in Figure 2. Response options were "yes" or" no".

**Table 2:** Four Health Care Systems: survey responses

***Outpatient care***	**Italy**	**Norway**	**Switzerland**	**UK**
				
Hours a week* (median, range)	12 (2–44)	33 (1–80)	40 (2–80)	12 (1–56)
Number of patients in half a day in clinic* (median, range)	11 (1–30)	10 (1–50)	12 (1–30)	15 (4–50)
Waiting time for an appointment* (median)	Within a week	Within two weeks	Next day	Within a month
				
***Inpatient care***				
Hours a week* (median, range)	35 (8–60)	20 (1–50)	14 (1–60)	24 (1–100)
Number of inpatients cared for at one time (median, range)	18 (3–150)	15 (2–82)	15 (1–270)	20 (1–85)
				
***Health system equity***	***Agree or Strongly agree***			
I am given enough means to treat my patients fairly *	65%	73%	81%	29%
Health resources in my country are distributed fairly*	35%	39%	69%	21%
Everyone in my country has equal access to needed medical services*	50%	36%	59%	11%

*Kruskall-Wallis: p < 0.01; null hypothesis is "no difference"

**Figure 3:**
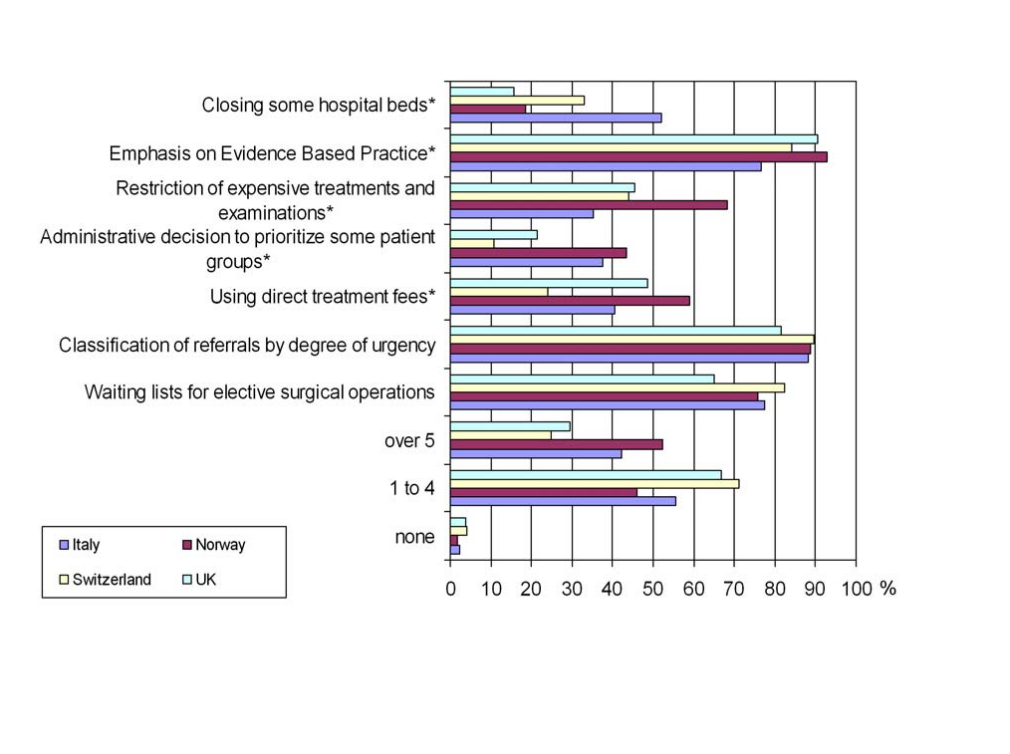
**Percentage of respondents who agreed with different cost-containment policies**. Based on your experience, how acceptable do you consider the following methods of resource allocation to be?. *Kruskall-Wallis: p < 0.01; null hypothesis is "no difference".

Physicians' attitudes towards cost-containment policies were explored using the items shown in Figure 3. Responses were on a 5 point Likert scale ranging from "not at all acceptable" (1) to "very acceptable" (5), with an additional option of "I have no experience with this".

We also gathered demographic information about respondents and their practice environment.

Participants were contacted by mail, and told about the aims of the study in a cover letter. Questionnaires were self-administered by the respondents. To maximize response rate, cover letters were addressed by local researchers affiliated with universities in the respondents' country. A repeat mailing was sent, including an additional copy of the questionnaire, and an incentive of €10, or the closest equivalent in local currency that could be enclosed as a single bill [21]. Questionnaires were mailed to 1600 physicians. Data collection was open from February 2003 to June 2004.

### Human subjects protection

Participation was voluntary and responses were made anonymous before analysis. Approval was given by the IRB of the National Institute of Child Health and Development at the US National Institutes of Health, and by the Trent Multi-Centre Research Ethics Committee in the UK. This study was examined and designated exempt from ethics committee review by IRBs in Norway, Italy, and Switzerland.

### Statistical analysis

Data were analyzed using descriptive statistics, and bivariate correlations were analyzed using Pearson Chi-square, Mann-Whitney, or Kruskall-Wallis tests as appropriate. We selected a significance level of 0.01 (two-tailed).

Logistic regression was used to identify variables independently associated with perceived scarcity, perceived equity, and perceived discrimination. The models were built using the variables that were found to be associated with these in bivariate analysis. We chose individual respondents, rather than countries, as our unit of analysis. This was based on the literature on practice variation, which shows availability of resources and utilization rates to vary geographically within a country [22], including in most of the countries we surveyed [23-25]. Consequently, we made the assumption of multiple micro-environments within countries, and chose individual respondents as more likely to reflect these multiple environments in our analysis.

## Results

### Respondents

Respondents, (N = 656, 43% of eligible sample) ranged in age from 28–82, and were predominantly male (85%), with the percentage of women ranging from 42.1% under the age of thirty to 7.8% from 61 to 70 years of age. The average length of time in practice was 25 years, and 38.4% were at least partly hospital-based. (Table 3)

**Table 3:** Respondent characteristics

	**Physicians (N = 656)**
	
**Characteristics**	
Age, years	28–82 (mean 51)
Years in practice	1–62 (mean 25)
Male	546 (85%)
Specialty	
Family medicine	195 (30%)
General medicine	188 (29%)
Internal medicine	179 (28%)
Country of practice	
Italy	139 (21%)
Norway	222 (34%)
Switzerland	183 (28%)
UK	112 (17%)
Primary practice site	
Hospital	258 (38%)
Solo practice	182 (28%)
Primary care group practice	164 (25%)
Multi-specialty group	23 (4%)
Other	28 (4%)
Admitting hospital	
Public	572 (94%)
Private	21 (3%)
For-profit	81 (17%)
Not-for-profit	406 (82%)
Teaching hospital	264 (46%)

Numbers in parentheses are percentages of the sample shown exclusive of missing data, and rounded to the nearest whole number

Respondents from different countries reported significantly different population density in their practice environments with the greatest percentage of physicians reporting rural environments in Norway (29%), and more reporting urban settings in Italy (49%) and the UK (38%) (p < 0.001). Maximum working hours in outpatient care ranged from 44 in Italy to 80 in Norway and Switzerland. (Table 2) Median number of patients seen in clinic, and waiting time for appointments, also differed significantly between the four surveyed countries. Maximum working hours in inpatient care ranged from 50 in Norway, to 100 in the UK.

### Scarcity

The vast majority of respondents (87.7%) perceived some resources as sometimes unavailable, with the most prominent being: access to nursing home, mental health services, referral to a specialist, referral to surgery, and rehabilitation for stroke (Figure 1). Mean score on the Perceived scarcity scale (range 0–44) was highest in the UK (9.4), followed by Italy (7.7), Norway (7.4), and Switzerland (4.2) (p < 0.001).

Perceived pressure to ration was reported to have occurred in the prior six months by 46.2% of respondents, with the greatest proportion in Switzerland (65%), followed by the UK (62.7%), Norway (47.7%, and (26.8%) (p < 0.001). Although all surveyed countries offer universal coverage, 45.6% of respondents reported instances where a medical problem could not be treated because patients could not afford their share of the cost. This was highest in Norway (58.9%), followed by Italy (50.4%), Switzerland (48%) and the UK (24.7%) (p < 0.001).

Most respondents (68%) reported adverse outcomes from scarcity, with this proportion lowest in Switzerland (55.3%), followed by Italy (64.2%), Norway (74.8%), and reaching 80% in the UK (p < 0.001). Respondents witnessed such adverse outcomes infrequently, with a median of less than once a month in all four countries. The most severe adverse event attributed to scarcity was described as an inconvenience by a third (30.5%) of respondents. However, a minority had encountered severe adverse events, such as death (16.5%) or permanent disability (7.2%). Others reported acute life-threatening events (11%), or temporary disability (14.7%).

In logistic regression, less scarcity was reported by respondents working in Switzerland (OR 0.4, 95% CI 0.2–0.8). Greater scarcity was reported by respondents who had witnessed adverse events attributed to scarcity (OR 1.5, 95% CI 1.2–1.9).

### Health care system equity

Mean score on the Perceived equity scale (range 3–15) was 9.5, with a high of 11.1 in Switzerland and a low of 7.2 in the UK (p < 0.001). While 92.8% of respondents thought everyone in their country should have equal access to needed medical services, 44.1% thought that health care resources in their country were not distributed fairly, 23.6% considered that they were not given enough means to treat their patients fairly, and 50.5% did not agree that everyone in their country had equal access to needed medical services (Table 2). In logistic regression, less equity was reported by physicians working in the UK, (OR 0.1, 95% CI 0.04–0.3), reporting more pressure to ration (OR 0.7, 95% CI 0.5–0.9), or who reported more adverse events attributed to scarcity (OR 0.7, 95% CI 0.5–0.9).

Mean scores on the Perceived discrimination index (range 0–8) were highest in Switzerland (3) and lowest in Italy (2.2) (p = 0.003). Most respondents (78.7%) reported that at least one group of patients was more likely than others to be denied beneficial care on the basis of cost in their health care environment. The most frequently identified groups were patients who are mentally incapacitated, patients who require chronic care, illegal immigrants, and patients who are old, respectively. There were significant differences between countries in the frequency with which each group was identified, except for legal immigrants and the elderly. (Figure 2) On logistic regression, more discrimination was reported by respondents who reported more underinsurance (OR 1.8, 95% CI 1.2–2.7), or more scarcity (OR 1.1, 95% CI 1.01–1.12). Less discrimination was reported by Italian physicians (OR 0.4, 95% CI 0.2–0.9).

### Cost containment policies

Almost all respondents (97.3%) found at least one cost-containment policy acceptable (Figure 3). Mean number of acceptable policies were 4, with a high of 4.5 in Norway, and a low of 3.7 in Switzerland and the UK (p < 0.001). Classification of referrals by degree of urgency, emphasis on evidence based practice, and waiting lists for elective surgery were the policies most frequently found acceptable. Administrative prioritization of patient groups and closing hospital beds were least frequently found acceptable, with the latter found acceptable more frequently (52%) in Italy (p < 0.001). Restriction of expensive treatments and interventions, and direct treatment fees, were found acceptable by over half of respondents only in Norway (68.2% and 58.9%, respectively) (p < 0.001). Overall agreement with cost-containment policies was greater in Norway and Italy than in the UK and Switzerland (p < 0.001). Agreement with cost-containment policies was not associated with perceived scarcity, equity, or discrimination, or with reporting adverse effects of scarcity.

## Discussion

Scarcity, or resource unavailability, was reported by physicians in all four surveyed countries. Despite universal coverage, physicians reported underinsurance. Serious consequences of scarcity were reported in all countries. Resource availability was unevenly distributed: some interventions were more frequently unavailable, and some patients were identified as more likely than others to be denied care on the basis of cost. Physicians, however, accepted cost-containment policies. They reported willingness to participate in cost-containment, and did not want to be guided by prioritization decisions made at an administrative level.

Our study has several limitations. It has been suggested that physicians often deny scarcity [2]. Although our results do not confirm this in the countries studied, physicians may still underestimate scarcity. There may also be pressures brought to bear on physicians, or expectations on the part of patients, but also physicians, that motivate them to think that more resources are necessary. This could lead to an overestimation of scarcity. However, as long as the interventions they consider to be indicated have at least marginal benefit, considering them to be unnecessary could be a matter of debate. As with all questionnaire studies, recall bias can be an issue. We used a conservative limit on the time we surveyed physicians about, however, they may still have remembered striking scarcity more than mundane everyday events [26]. This could lead to an underreporting of scarcity, and a relative overreporting of the more serious kind of resource unavailability. Regarding the availability of specific resources, responses about mental health and chronic care bed shortages do seem to have face validity [27,28]. Asking about the most serious adverse event they had encountered in the previous six months, rather than the most frequent, may also mean that recall bias could be a lesser concern on that specific item. As we only surveyed general physicians, generalizations to other medical specialties, or to other health care systems should be cautious. Our results are also limited to the availability of resources to patients who have reached a physician in the first place. Finally, the response rate was modest, as is often the case for physicians [29] and questionnaires addressing sensitive topics [21]. Non-respondent bias is most likely to be associated either with lack of time, or with lack of interest with the topic. The latter could have led to an overestimation of scarcity, with a response bias in favor of physicians who were concerned with this problem. Reluctance to report an adverse impact on patient care could also have led to underreporting of scarcity and scarcity-related adverse events. However, extrapolating our results to a response rate of 100%, and considering all non-respondents to report no scarcity still results in a percentage of physicians reporting scarcity of 36%. One concern could be that the associations between variables could be affected by non-response bias. Variables independently associated with reported scarcity were reporting adverse events related to scarcity, and reporting less equity or more discrimination. If non-response were due primarily to lack of interest in the topic, then we could expect overestimation of adverse events related to scarcity, as well as overestimation of discrimination and lack of equity. As this would also likely be associated with overestimation of reported scarcity, however, the association between these two variables may not be affected.

Reports of scarcity in all the surveyed health care systems is not surprising. Every system in the world rations health care, some by wait times, some by availability of services, coverage decisions, or by ability to pay. There are thus good reasons for some resources to be unavailable, as choices will have to be made whenever demands exceed resources. Physicians are in a unique position to observe the impact of these choices, including when they may be unexpected. Our respondents' aggregate assessment of how various interventions were more or less sufficiently available differed across interventions, and between countries. Health care systems do not allocate their resources in identical ways; assessement of how existing services fit with perceived need, however, can be difficult. Despite growing research on variations in the distribution of resources in health care systems, and in utilization [22], there is no gold standard on the proper availability of resources. Utilization is often used as a proxy outcome for availability, but making the distinction between utilization, need, and availability can be challenging [30]. Availability is thus difficult to evaluate [31]. In our study, we assessed unavailability of services based on physicians' assessment of need rather than on a measure derived from utilization. Physicians' situation at the point of care enables them to perceive discrepancies between need, and utilization, that may begin to serve as a more precise description of the actual availability of services. Their view may also contribute to an understanding of what a reasonable level of resources, or a more appropriate level, ought to be. Our results thus provide insights into the impact of different health care systems, with different structures and expenditures on health care. Mean scores on the scarcity scale were consistent with differences in national health expenditures. Where comparisons are possible, physician reports of scarcity based on our findings are supported by OECD mortality data, which yields identical rankings with regard to colon cancer screening and mental health services, and an almost identical ranking regarding rehabilitation for stroke (Table 4) [32].

**Table 4:** Differences in reported unavailability is parallel to health outcomes

	**Italy**	**Norway**	**Switzerland**	**UK**
				
% respondents who reported unavailable rehabilitation for stroke^a^	57	53	23	44
Potential years of life lost, cerebrovascular disease/100,000 p. >70 years^b^	89	74	58	121
				
% respondents who reported unavailable colon cancer screening^a^	28	29	8	27
Potential years of life lost, malignant neoplasia of the colon/100,000 p. >70 years^b^	73	89	56	70
				
% respondents who reported unavailable mental health services^a^	37	80	58	53
Potential years of life lost, mental disorders/100,000 p. >70 years^b^	33	267	132	113

a Survey responses

b OECD 2002–3 country information

It would clearly be exaggerated to draw from this the conclusion that scarcity is the major cause of the differences in mortality reported here. For example, an alternative interpretation could be that physicians are more aware of problems related to diseases that are more prevalent. These comparisons, however, give construct validity to differences in the perception of scarcity between the four countries. If physicians were reporting different degrees of scarcity for, say, cultural reasons, we would not expect scarcity and disease-related mortality to be so parallel.

Although physicians's perception will be limited to situations where patients have reached them in the first place, their perception of scarcity may help to assess availability, a crucial element of access to health care [31].

Despite universal coverage, physicians reported underinsurance. This should not be surprising. Universal health insurance means that coverage extends to all persons who legally reside in the country, as well as to foreigners in situations of emergency. It does not, however, necessarily mean that access to all interventions will be covered financially. For example, Switzerland and Norway mostly do not include coverage for dental care in health insurance. Neither does it mean that all included interventions will be covered without cost-sharing. As shown in table 1, this factor can vary extensively between the four systems. The extent of reported underinsurance was not related to the amount of national health care expenditure, suggesting that organizational factors and coverage decisions also contribute to apparent underinsurance.

Adverse outcomes attributed to scarcity were witnessed by most physicians, if infrequently. Some reported severe outcomes, such as death. This is concerning and warrants further research. However, it must be noted that we lack sufficient detail regarding the specific cases to formulate a judgment regarding the accuracy of this attribution, or its comparability across health care systems. The association between scarcity and reported adverse events may signify true lack of necessary resources. When extrapolated to the population served by general physicians, the estimate based on our respondents' report yields 0.15 scarcity-related deaths/1000 population [33]. This is the same as the lower estimate, and 44% of the higher estimate, for deaths due to medical errors in the U.S [34]. It may, however, also suggest greater sensitivity in the perception of scarcity by physicians who have been confronted with a possible adverse outcome. Either way, physicians reporting death as an outcome of scarcity are likely to be dissatisfied either with the level of resources in their health care system, with its distribution, or both.

Access was often reported as less than equal. More specifically, some patient groups were identified as more likely than others to be denied care on the basis of cost. Although the WHO distribution of responsiveness was identical in the four studies countries, Perceived equity was different in different countries, as was Perceived discrimination. Respondents thus perceived that access, viewed as a concern that "health care resources are mobilized to meet the needs of different groups in the population" [35] was not fully realized. Respondents' views about equity did not vary in the same way as their views about discrimination did. Although physicians may be judging equity by standards different from the ones offered in our survey, a more likely explanation is that specific questions about patient groups were more likely to bring real cases to their minds. Thus, perceived discrimination may be a more sensitive tool to assess fairness in the distribution of health care resources. This finding also suggests that physicians, who are in a unique position to observe unequal access or discrimination in the health system, should be better equipped to address it. It is relatively easy for persons in a health care system to express a need for more resources but it is more difficult to develop an allocation process to ensure equitable distribution and resources allocated to a place to maximize benefit in terms of organizational or system objectives. Could physicians contribute to this? Data suggest that concerns for fairness are rarely explicit when physicians manage scarcity [3]. More explicit thinking about fairness, and perhaps specific training, could enable physicians to make therapeutic decisions that enhance equitable access to medical resources. Concerns for fairness are applicable to clinical practice [36]. In applying frameworks for fair resource allocation, implementing mechanisms for appeal and revisions [37] would also give practitioners the opportunity to bring experience from clinical practice to bear on prioritization. Furthermore, our results suggest that efforts to measure a health system's equity might incorporate feedback from physicians about adverse events stemming from distributional decisions made at the system level. This feedback loop could be a way to connect the macro and meso levels of priority setting with the micro level.

Comparisons with other assessments of equity and utilization show some convergence. An OECD working paper evaluated General Practitioner care utilization to be pro-poor in all four countries included in our study, but specialist utilisation to be pro-rich in all of them [38]. Reports by general physicians in our study that patients who cannot afford to pay for treatment are more likely to be denied care fits with those results. The degree of pro-rich inequity assessed by van Doorslaer and colleagues was highest in Italy, and lowest in the UK.

Our results suggest a link between perceived scarcity and perceived equity. Less equity was reported by physicians who attributed adverse events to scarcity, or more pressure to ration. More discrimination was perceived by those who reported more underinsutance or scarcity. This could mean that when there is less the most vulnerable are the first to get less. This view is both plausible and concerning.

Overall, however, physicians accepted cost-containment. Our results thus confirm that physicians are not fundamentally averse to such policies [10,14,39]. Indeed, support was greater in our sample than in the study initially using the items we included [14]. Respondents also indicated willingness to participate in these decisions: cost-containment policies close to the bedside were the most frequently approved. This suggests that physicians are not only ready to recognize that cost should play a role in allocating health care resources, but would rather participate in this sort of decision than not. If they are attentive to issues of fairness, they may be well situated to promote fair access to services in the face of resouce constraints.

## Conclusion

Physicians reported significantly different levels of resource availability, perceived health care equity, and discrimination, in Italy, Norway, Switzerland, and the UK. In the face of scarcity, and despite scarcity-related adverse events, physicians accepted cost-containment policies, and were willing to participate in cost-containment decisions. While one might expect fewer perceptions of underinsurance and discrimination among physicians in countries with greater health care expenditure, this was not the case, suggesting that organizational factors and allocations decisions in the health care system may have an effect as well. If they are attentive to issues of fairness, physicians may be well situated to promote fair access to services even in the face of resouce constraints. Tools should be developed to enable physicians, who are in a unique position to observe unequal access or discrimination in their health care environment, to address these issues in a more targeted way. Results from the four countries studied here, all of which provide universal health insurance, may serve as a benchmark for studies in other countries.

## Competing interests

The author(s) declare that they have no competing interests.

## Authors' contributions

All authors contributed to the conception of this paper, and to the acquisition of data. SAH wrote the first draft and all authors made important contributions to subsequent drafts. All authors have seen and approved the final version. SAH and MD had full access to all of the data in the study and take responsibility for the integrity of the data and the accuracy of the data analysis.

## Pre-publication history

The pre-publication history for this paper can be accessed here:



## References

[B1] Aaron HJ, Schwartz WB (1984). The Painful Prescription; Rationing Hospital Care. Studies in Social Economics.

[B2] Alexander GC, Werner RM, Ubel PA (2004). The costs of denying scarcity. Arch Intern Med.

[B3] Hurst SA, Hull SC, DuVal G, Danis M (2005). Physicians' responses to resource constraints. Arch Intern Med.

[B4] Baines DL, Tolley KH, Whynes DK (1998). The ethics of resource allocation: the views of general practitioners in Lincolnshire, U.K. Soc Sci Med.

[B5] Ryynanen OP, Myllykangas M, Kinnunen J, Takala J (1997). Doctors' willingness to refer elderly patients for elective surgery. Fam Pract.

[B6] Wouters MW, Timmermans DR, Kievit J (1997). [Financial limits to care; a survey among physicians]. Ned Tijdschr Geneeskd.

[B7] Ryynanen OP, Myllykangas M, Kinnunen J, Halonen P, Takala J (2000). Prioritization attitudes among doctors and nurses examined by a scenario method. Int J Technol Assess Health Care.

[B8] Ubel PA, Baron J, Nash B, Asch DA (2000). Are preferences for equity over efficiency in health care allocation "all or nothing"?. Med Care.

[B9] Cooke L, Hutchinson M (2001). Doctors' professional values: results from a cohort study of United Kingdom medical graduates. Med Educ.

[B10] Rosen P, Karlberg I (2002). Opinions of Swedish citizens, health-care politicians, administrators and doctors on rationing and health-care financing. Health Expect.

[B11] Perneger TV, Martin DP, Bovier PA (2002). Physicians' attitudes toward health care rationing. Med Decis Making.

[B12] van Delden JJ, Vrakking AM, van der Heide A, van der Maas PJ (2004). Medical decision making in scarcity situations. J Med Ethics.

[B13] Escher M, Perneger TV, Chevrolet JC (2004). National questionnaire survey on what influences doctors' decisions about admission to intensive care. Bmj.

[B14] Ryynanen OP, Myllykangas M, Kinnunen J, Takala J (1999). Attitudes to health care prioritisation methods and criteria among nurses, doctors, politicians and the general public. Soc Sci Med.

[B15] Arnesen T, Fredriksen S (1995). Coping with obligations towards patient and society: an empirical study of attitudes and practice among Norwegian physicians. J Med Ethics.

[B16] Hurst SA, Slowther AM, Forde R, Pegoraro R, Reiter-Theil S, Perrier A, Garrett-Mayer E, Danis M (2006). Prevalence and Determinants of Physician Bedside Rationing: Data from Europe. J Gen Intern Med.

[B17] van Kammen J, de Savigny D, Sewankambo N (2006). Using knowledge brokering to promote evidence-based policy-making: The need for support structures. Bull World Health Organ.

[B18] Hurst SA, Forde R, Reiter-Theil S, Pegoraro R, Perrier A, Slowther A, Danis M (2007). Ethical Difficulties in Clinical Practice: Experiences of European Doctors. Journal of Medical Ethics.

[B19] Sulmasy DP, Bloche MG, Mitchell JM, Hadley J (2000). Physicians' ethical beliefs about cost-control arrangements. Arch Intern Med.

[B20] DuVal G, Clarridge B, Gensler G, Danis M (2004). A national survey of U.S. internists' experiences with ethical dilemmas and ethics consultation. J Gen Intern Med.

[B21] Edwards P, Roberts I, Clarke M, DiGuiseppi C, Pratap S, Wentz R, Kwan I (2002). Increasing response rates to postal questionnaires: systematic review. Bmj.

[B22] Wennberg JE, MacAndrew Cooper M (1999). The Quality of Medical Care in the United States: a Report on the Medicare Program; the Dartmouth Atlas of Health Care 1999.

[B23] Carlsen F, Grytten J (1998). More physicians: improved availability or induced demand?. Health Econ.

[B24] Crivelli L, Domenighetti G (2003). [The physician/population ratio in Switzerland: the impact of its regional variation on mortality, health expenditures and user's satisfaction]. Cah Sociol Demogr Med.

[B25] Price CE, Paul EA, Bevan RG, Holland WW (1992). Equity and medical practice variation: relationships between standardised discharge ratios in total and for selected conditions in English districts. J Epidemiol Community Health.

[B26] Ubel PA (2006). Tough questions, even harder answers. J Gen Intern Med.

[B27] Knapp M, Funk M, Curran C, Prince M, Grigg M, McDaid D (2006). Economic barriers to better mental health practice and policy. Health Policy Plan.

[B28] Netten A, Darton R, Williams J (2003). Nursing home closures: effects on capacity and reasons for closure. Age Ageing.

[B29] Asch DA, Jedrziewski MK, Christakis NA (1997). Response rates to mail surveys published in medical journals. J Clin Epidemiol.

[B30] Andersen R, Aday LA (1978). Access to medical care in the U.S.: realized and potential. Med Care.

[B31] Penchansky R, Thomas JW (1981). The concept of access: definition and relationship to consumer satisfaction. Med Care.

[B32] OECD (2006). OECD Health Data; Statistics and indicators for 30 countries.

[B33] OECD Health Data 2005. http://www.oecd.org/document/16/0,2340,en_2649_34631_2085200_1_1_1_1,00.html.

[B34] Kohn LT, Corrigan JM, Donaldson MS, Press NA (1999). To err is human; building a safer health care system.

[B35] Gulliford M, Figueroa-Munoz J, Morgan M, Hughes D, Gibson B, Beech R, Hudson M (2002). What does 'access to health care' mean?. J Health Serv Res Policy.

[B36] Hurst SA, Danis M (2007). A Framework for Rationing by Clinical Judgment. Kennedy Inst Ethics J.

[B37] Daniels N (2000). Accountability for reasonableness. Bmj.

[B38] van Doorslaer E, Masseria C, Koolman X (2006). Inequalities in access to medical care by income in developed countries. Cmaj.

[B39] Myllykangas M, Ryynanen OP, Kinnunen J, Takala J (1996). Comparison of doctors', nurses', politicians' and public attitudes to health care priorities. J Health Serv Res Policy.

[B40] WHO Countries: selected indicators. http://www.who.int/whosis/database/core/core_select_process.cfm.

[B41] WHO (2000). World Health Report; Health Systems: Improving Performance.

